# Overexpressing *Arabidopsis thaliana ACBP6* in transgenic rapid-cycling *Brassica napus* confers cold tolerance

**DOI:** 10.1186/s13007-022-00886-y

**Published:** 2022-05-12

**Authors:** Aruni Y. Alahakoon, Eden Tongson, Wei Meng, Zi-Wei Ye, Derek A. Russell, Mee-Len Chye, John F. Golz, Paul W. J. Taylor

**Affiliations:** 1 Faculty of Veterinary and Agricultural Sciences, The University of Melbourne, Parkville, VIC Australia; 2 School of Biological Sciences, The University of Hong Kong, Pokfulam, Hong Kong China; 3 School of BioSciences, The University of Melbourne, Parkville, VIC Australia

**Keywords:** Acyl-CoA-binding proteins, *Arabidopsis thaliana ACBP6*, *Brassica napus*, *B. napus-*RC cold tolerance, Freezing tolerance, Frost tolerance, In vitro regeneration, Rapid-cycling *Brassica*, Transformation

## Abstract

**Background:**

Rapid-cycling *Brassica napus* (*B. napus*-RC) has potential as a rapid trait testing system for canola (*B. napus*) because its life cycle is completed within 2 months while canola usually takes 4 months, and it is susceptible to the same range of diseases and abiotic stress as canola. However, a rapid trait testing system for canola requires the development of an efficient transformation and tissue culture system for *B. napus*-RC. Furthermore, effectiveness of this system needs to be demonstrated by showing that a particular trait can be rapidly introduced into *B. napus*-RC plants.

**Results:**

An *in-vitro* regeneration protocol was developed for *B. napus*-RC using 4-day-old cotyledons as the explant. High regeneration percentages, exceeding 70%, were achieved when 1-naphthaleneacetic acid (0.10 mg/L), 6-benzylaminopurine (1.0 mg/L), gibberellic acid (0.01 mg/L) and the ethylene antagonist silver nitrate (5 mg/L) were included in the regeneration medium. An average transformation efficiency of 16.4% was obtained using *Agrobacterium*-mediated transformation of *B. napus*-RC cotyledons using *Agrobacterium* strain GV3101 harbouring a plasmid with an *NPTII* (kanamycin-selectable) marker gene and the *Arabidopsis thaliana* cDNA encoding ACYL-COA-BINDING PROTEIN6 (AtACBP6). Transgenic *B. napus*-RC overexpressing *AtACBP6* displayed better tolerance to freezing/frost than the wild type, with enhanced recovery from cellular membrane damage at both vegetative and flowering stages. *AtACBP6*-overexpressing *B. napus*-RC plants also exhibited lower electrolyte leakage and improved recovery following frost treatment, resulting in higher yields than the wild type. Ovules from transgenic *AtACBP6* lines were better protected from frost than those of the wild type, while the developing embryos of frost-treated *AtACBP6*-overexpressing plants showed less freezing injury than the wild type.

**Conclusions:**

This study demonstrates that *B. napus*-RC can be successfully regenerated and transformed from cotyledon explants and has the potential to be an effective trait testing platform for canola. Additionally, AtACBP6 shows potential for enhancing cold tolerance in canola however, larger scale studies will be required to further confirm this outcome.

**Supplementary Information:**

The online version contains supplementary material available at 10.1186/s13007-022-00886-y.

## Background

*Brassica napus* L. (canola/rapeseed) accounts for the second largest production of oilseed globally after soybean [1] and its seeds are used to extract a healthy edible oil, while the crushed seeds after oil extraction provide a good source of protein for livestock [2]. Additionally, there is demand for the oil for industrial purposes and as a biodiesel [1]. As a result, the global production of rapeseed has increased by almost three-fold over the last three decades, reaching 70 million metric tonnes in 2019 [3]. In the field, canola production is challenged by various biotic and abiotic stresses. Low temperature stress, including frost, leads to severe financial losses for the canola industry in temperate countries [4, 5]. Transgenic technologies have the potential to ameliorate the effects of these environmental and biological constraints, by introducing foreign genes that confer stress tolerance [6–8]. Genetic engineering has provided opportunities for testing a range of traits to enhance crop performance in species in which it would be impossible to achieve by conventional breeding [9].

Westar, a model cultivar of canola, can take about 4 months to complete its lifecycle under optimal conditions in the greenhouse. The use of a model system with a much shorter lifecycle would allow rapid testing of traits in a similar genetic background as the targeted crop. The first steps toward developing such a potential *Brassica* model species were taken by recurrent selection of early flowering lines of *Brassica* species obtained from the United States Department of Agriculture’s National Germplasm System [10]. This resulted in the development of short generation (rapid-cycling, RC) populations for six important Brassicaceae species, *B. rapa, B. oleracea, B. juncea, B. carinata, B. nigra* and *B. napus* [10] which can complete their seed-to-seed life cycle within 2 months*.* Rapid-cycling plants have traits that make them ideal models, such as relatively small size, high female fertility, lack of seed dormancy, easy pollination, ability to grow under 24 h fluorescent light in standard potting mix and rapid seed maturity [11, 12]. These characteristics assist in more rapidly testing novel traits when compared to canola varieties. Importantly, *B. napus*-RC populations succumb to the same environmental stresses and diseases as canola [13, 14], which potentially would make these plants an efficient trait testing system for canola. Although rapid-cycling *Brassicas* have been regenerated from protoplasts [15, 16], anthers [12], and seedlings using somatic embryogenesis [17], hypocotyls and cotyledons [18, 19] regeneration frequencies need to be optimised for development of a practical transformation system.

*Agrobacterium*-mediated transformation is an efficient gene transfer system for *Brassica napus* [20, 21]. Regeneration of *B. napus* using tissue-culture based genetic transformation is highly dependent on the explant type [22], genotype [21, 23], age, growth media composition [24] and effective antibiotic selection of transformants [20] as well as the *Agrobacterium* strain and bacterial concentration [25]. Spring type *B*. *napus* varieties e.g. Westar, are considered more amenable to genetic transformation than the winter types [20]. The explant types most frequently used in canola *B. napus* transformation have been cotyledons [25–27] and hypocotyls [22, 23, 28–30]. Explants derived from young seedlings are more responsive to regeneration in tissue culture media than those from older plants [31]. Cotyledon explants are generally prepared from 4 to 6-day-old seedlings [32] while hypocotyl explants are from 5- to 7-day-old seedlings [8].

Poor regeneration efficiencies recorded in RC-*Brassica* experiments [12, 15–19] may have been a reason for the lack of a detailed tissue culture-based transformation protocol for *B. napus*-RC. A necessary first step towards transformation of rapid-cycling plants is to test plant regulator concentrations and combinations derived from published protocols for canola [25, 28] to ascertain whether these are appropriate for the transformation of rapid-cycling *Brassicas* and then to optimise them if necessary. Establishing an efficient transformation protocol for rapid-cycling *Brassicas* will facilitate high-throughput testing of transgenes in *B. napus*-RC instead of in canola*,* thus, reducing labour, time, costs and facility requirements.

Cold stress and frost injury are common abiotic stresses affecting canola and other seed crops in the temperate regions. These stresses can cause severe effects on the development and growth of plants [33, 34], including death. Australia has significant risks of frost over large areas, especially in the southern regions of the country. Such frost events lead to losses across all crops of 120 to 700 million Australian dollars annually to the agricultural industry [35]. Some proteins such as the *Arabidopsis thaliana* ACYL-COA-BINDING PROTEIN6 (AtACBP6), a small (10 kDa) cytosolic protein, have been reported to confer cold tolerance in transgenic *A. thaliana* when overexpressed [36–38]. Consistent with these genes being associated with cold-tolerance, *AtACBP6* overexpression was shown to be induced by cold exposure at 4 °C for 48 h [36] while *atacbp6* mutant plants were susceptible to freezing temperatures [36]. Thus, the overexpression of *AtACBP6* in *B. napus* may provide tolerance to cold/frost. This paper describes the development of a genetic transformation system for *B. napus*-RC plants, and the effects of overexpression of the test trait *AtACBP6* in conferring cold tolerance.

## Results

### In vitro regeneration from cotyledons and hypocotyls of *B. napus*-RC

**Fig. 1:**
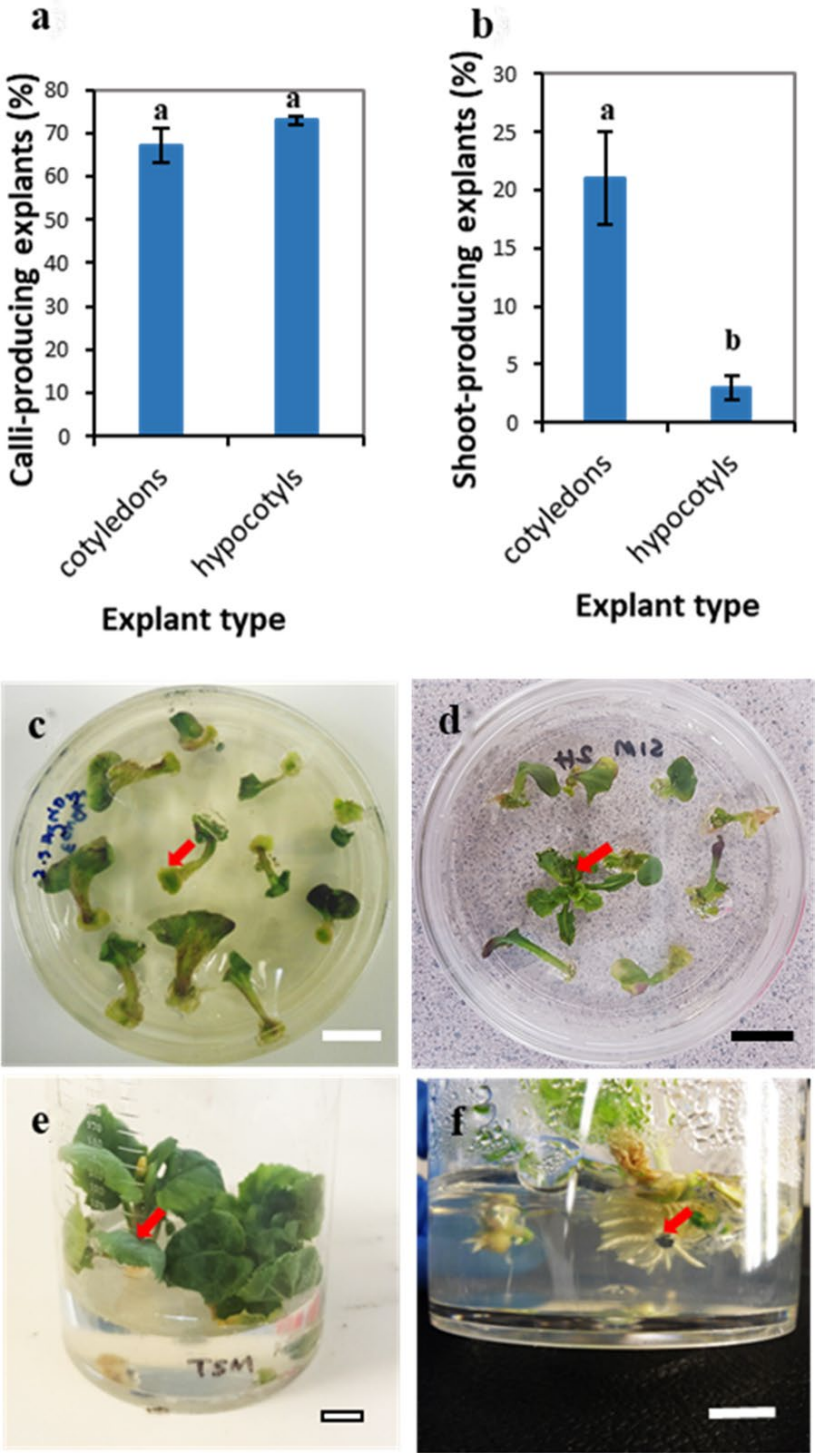
Efficiency of *B. napus*-RC cotyledons and hypocotyls in producing calli and shoots in vitro. Proportion of *B. napus*-RC cotyledons and hypocotyls producing calli **a** and shoots **b**, in tissue culture media containing plant growth regulators NAA (0.1–0.3 mg/L), BAP (0.5–1.5 mg/L), GA_3_ (0.01 mg/L) and the ethylene antagonist, AgNO_3_ (5 mg/L)_._ The error bars represent standard error of mean; means that do not share the same letter are significantly different from each other according to Fisher’s Least Significance Difference (protected) test at the 5% confidence level (n = 10, r = 18). **c** Representative calli (arrow) arising from cotyledon petioles following incubation on callus induction medium. **d** Representative shoots (arrow) from cotyledons on shoot induction medium where small shoots/plantlets bud off from developed calli. **e** Elongation of shoots (arrow) from hypocotyls on shoot elongation medium. **f** Root intitials (arrow) from plantlets in root induction medium. Scale bar, 1 cm

Calli were initiated on cotyledon explants after 3–4 days in callus induction medium (CIM) and were prominent after one week. When hypocotyls were used as the explant, callus induction took longer (1–2 weeks in CIM) with calli not readily apparent before two weeks in the media (Additional file 1: Fig. S1a, b). At 2-weeks, 67% of cotyledon and 73% of hypocotyl explants produced calli with no significant difference (p > 0.05) between explant type (Fig. 1a). Calli on the cotyledon explants started to produce small leaf primordia (mean 21% n = 10; Fig. 1b) within the first week in shoot induction medium (SIM). During the following week, leaf primordia developed further to bear 2–3 true leaves per plantlet (Additional file 1: Fig. S1c). In contrast, calli on hypocotyl explants took at least 4 weeks to induce shoots and had a lower proportion of explants with shoot initials (mean success 3%, 18 plates, 10 calli/plate: Fig. 1b, Additional file 1: Fig. S1d). These were smaller than the shoots that developed from cotyledon explants and displayed malformation, vitrification and hyperhydricity (succulence/watery appearance) (Additional file 1: Fig. S1d). Shoot induction from cotyledons (mean 21%) was significantly higher than from hypocotyls (Fig. 1b).

### Optimisation of callus induction and shoot induction from cotyledons

The five different CIM treatments (Additional file 1: Table S6) were equally effective in the initiation of callus tissue (88–97%) from rapid-cycling cotyledon explants, whereas the no-treatment control was unable to produce healthy calli (Additional file 1: Fig. S2a). Shoot induction from cotyledon explants significantly increased with the addition of the ethylene antagonist, silver nitrate (AgNO_3_) to the SIM, with the percentage of explants producing calli showing shoot induction between 58 and 75% (Additional file 1: Fig. S2b). Only 30% of explants producing calli were successful in producing shoots in absence of AgNO_3_. The highest average shoot regeneration percentage was achieved from 4-day-old cotyledons when the SIM was supplemented with NAA (0.1 mg/L), BAP (1.0 mg/L), gibberellic acid (GA_3_) (0.01 mg/L) and AgNO_3_ (5 mg/L) (Additional file 1: Fig. S2c, treatment II). However, increasing the concentration of BAP, a well-known cytokinin which promotes shoot growth, to 5 mg/L while keeping the other PGRs constant (Additional file 1: Table S7 treatments III and IV) did not improve shoot production (Additional file 1: Fig. S2c). BAP (4 mg/L) coupled with zeatin (2 mg/L) (Additional file 1: Table S7, treatments V and VI) also did not further enhance shoot induction (Additional file 1: Fig. S2c).

### Optimising root induction

In total, sixty-five per cent of the shoots derived from cotyledons produced roots in root induction medium (RIM) supplemented with IBA at 2 mg/L, a slightly higher proportion than when IBA was used at 1 mg/L and considerably better than at 0.5 mg/L or without the use of IBA (Additional file 1: Fig. S2d).

### Transformation of *B. napus*

**Fig. 2:**
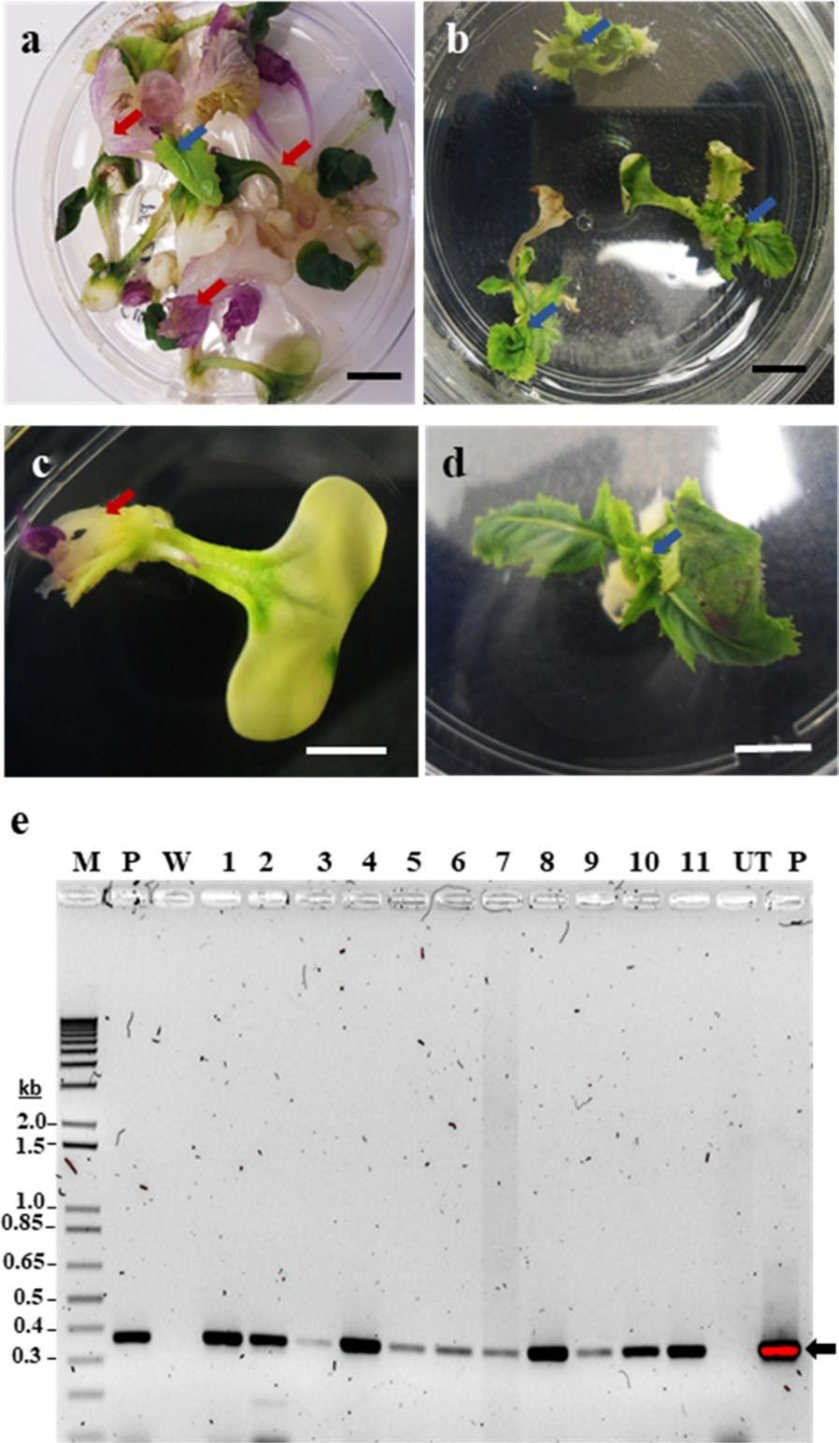
Generation of *AtACBP6*-overexpressing *B. napus*-RC shoots cultured in vitro on medium containing kanamycin (25 mg/L) applied two weeks after transformation to differentiate transformed from untransformed shoots; and subsequent PCR screening. **a** On selective shoot media, non-transgenic shoots are pink or pale-yellow (red arrows) whereas transgenic shoots are green (blue arrow). **b** Putative transformed green shoots (arrows). **c** Close-up of representative yellow cotyledon explant with an untransformed purple shoot (arrow). **d** Close-up of a cotyledon explant with a putatively transformed green shoot (arrow). Black scale bar, 1 cm; white scale bar, 5 mm. **e** PCR screening of regenerated shoots using an *AtACBP6*-specific primer pair ML838 and ML750. The expected amplicon size (0.37 kb) is arrowed. M, 1 kb plus DNA ladder; P, plasmid pAT593 positive control; W, water control; Putative transformed green shoot samples, 1–11; UT, untransformed *B. napus*-RC

Initial transformation experiments with cotyledons using plasmid pAT593 (Additional file 1: Fig. S3) containing CaMV *35S::AtACBP6* were conducted by preparing the explants in Murashige & Skoog (MS) [39] liquid media. The antibiotic selection was applied a week after the co-cultivation step. This method resulted in very low production of putatively transformed kanamycin-resistant shoots. The average proportion of explants producing green shoots in kanamycin media and subsequently confirmed to be transformed, using PCR, was between 2 and 5% (Additional file 1: Table S2, Fig. 2a, c). However, changes in the procedure involving a delay in the initial antibiotic selection by two weeks allowed improved transgenic cell proliferation and establishment. The elimination MS liquid media from the explant preparation surface, and immediate dipping in the *Agrobacterium* solution, significantly enhanced (p < 0.05) putative transformed shoot production in subsequent transformation experiments using cotyledons, with an average transformation efficiency of 16.4% in 3 independent experiments (Table 1, Additional file 1: Table S3). Two rounds of selection pressure (25 mg/L and 50 mg/L of kanamycin) applied at the shooting stage starting at 2-weeks after transformation rather than 1-week, ensured that transgenic plants had developed sufficiently to withstand the selection pressure and, thus, reduced the loss of putative transgenic lines (Fig. 2b, d, Additional file 1: Table S3).

**Table 1:** Comparison of transformation efficiencies of *B. napus*-RC in initial experiments which used MS liquid in cotyledon preparation and applied selection 1 week after co-cultivation, with subsequent experiments where explants were prepared without liquid and selection was applied 2 weeks after co-cultivation

MS liquid in explant preparation medium	Antibiotic selection after co-cultivation	Mean % explants producing PCR confirmed transformed shoots
Y	1 week	4.90^**b**^
N	2 weeks	16.5^**a**^

Means that do not share the same letter are significantly different from one another (p < 0.05) according to Fisher’s Least Significance Difference (protected) test (n = 3 independent experiments)

### Verification of *AtACBP6* lines

**Fig. 3:**
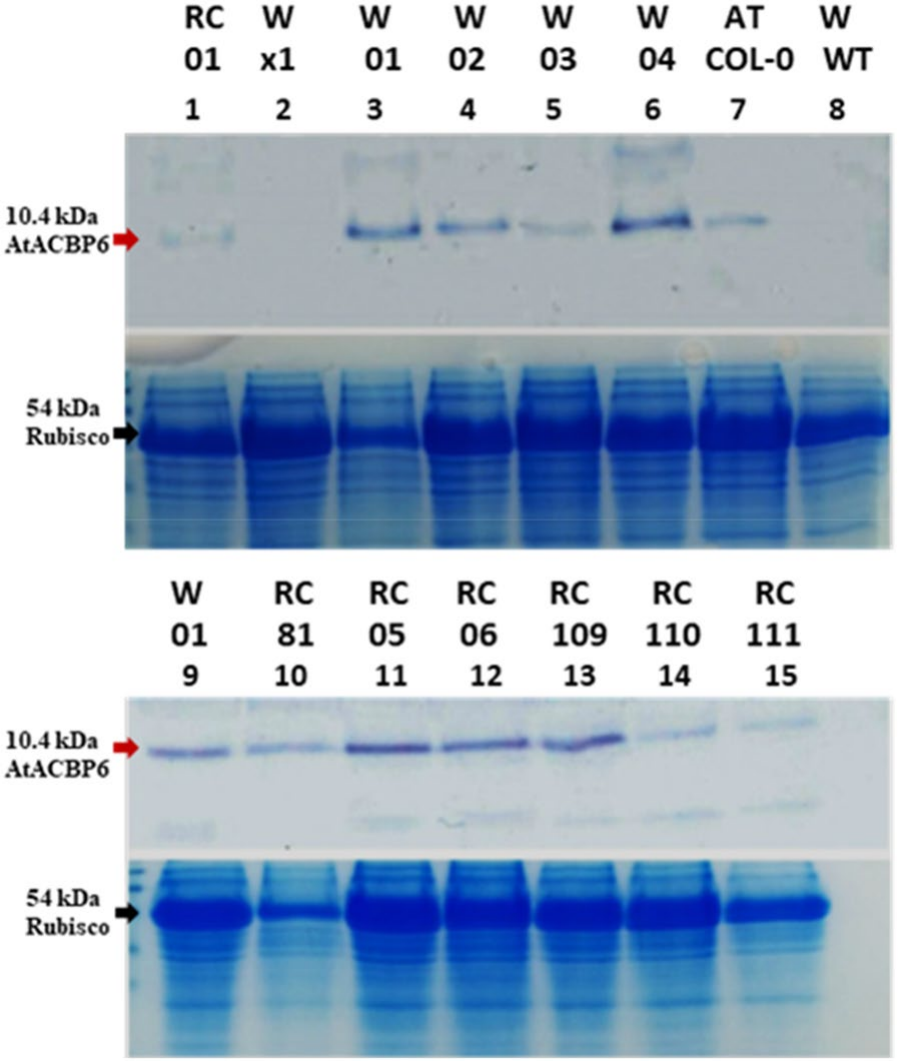
Detection of AtACBP6 protein in transgenic *B. napus*-RC and Westar lines by western blot analysis with AtACBP6 specific antibodies. The top half of each panel shows the protein blot with 10.4-kD AtACBP6 cross-reacting band (red arrow), and the bottom panel shows an identically loaded gel stained with Coomassie blue. Lane 1, *AtACBP6*-overexpressing *B. napus*-RC (RC) line 01; Lanes 2–6, *AtACBP6*-overexpressing Westar (W) lines × 1, 01, 02, 03 and 04; Lane 7, *A. thaliana* (AT) Col-0; Lane 8, wild-type *B. napus* (WT); Lane 9, *AtACBP6*-overexpressing Westar line 05; Lane 10 to 15, transgenic *B. napus*-RC plants lines 81, 05, 06, 109, 110, 111

T_0_ transgenic Westar and *B. napus*-RC lines over-expressing *AtACBP6* (Westar lines: 01, 02, 03, 04, 05 and rapid-cycling lines: 01, 81, 109, 111) generated by *Agrobacterium*-mediated transformation of cotyledon explants were confirmed by PCR (Additional file 1: Fig. S4a, b, Additional file 1: Table S10), semi-quantitative PCR (Additional file 1: Fig. S4c) and Western blot analysis (Fig. 3). PCR analysis using 35SB/ML838 primers generated an amplicon of size of 0.4 kb from each of these selected transgenic lines, corresponding to a region spanning *CaMV35S* and part of the *AtACBP6* coding sequence (Additional file 1: Fig. S4a). PCR with primers NPTII-2F/NPTII-2R also revealed the presence of the selectable marker *NPTII* in these lines through detection of a 0.7-kb fragment corresponding to the plasmid pAT593 (Additional file 1: Fig. S4b). Subsequently, reverse transcription RT-PCR (Additional file 1: Fig. S4c) using *AtACBP6*-specific primers ML750/ACBP02 produced the expected 0.37-kb amplicon, confirming expression of *AtACBP6* mRNA in all putatively transformed rapid-cycling lines 01, 81, 109, 111 and Westar lines 01, 02, 03, 04, 05. Western blot detected a cross-reacting AtACBP6 protein band (10.4 kDa) in each of these *AtACBP6*-overexpressing lines confirming presence of the AtACBP6 protein (Fig. 3).

### Cold tolerance and recovery in *B. napus-*RC lines

#### Electrolyte leakage

Conductivity measurements of leaf discs revealed a lower conductance and so a lower electrolyte leakage in rapid-cycling *AtACBP6*-overexpressing lines 01, 81 and 109 compared to wild type at the vegetative, flowering, early podding and seed-setting stages when cold challenged (Table 2). Line 111 had a significant reduction in conductance at the vegetative stage but not at the flowering stage. Frost-treated *AtACBP6*-overexpressing vegetative stage plants showed less than half the electrolyte leakage of the wild-type controls (Table 2). The percentage electrolyte leakage reported for frost-treated flowering plants was between 61 to 70% in transgenic lines and significantly higher at 81% in the controls, suggesting higher membrane damage in all plants at flowering than at other developmental stages (Table 2). The percent electrolyte leakages of early-podding staged plants ranged between 51 to 60% in rapid-cycling *AtACBP6*-overexpressing plants in comparison to 96% in the wild type (Table 2). At the seed-setting stage, frost-treated *AtACBP6* plants showed significantly lower electrolyte leakage (33–45%) than the wild type (69%) (Table 2).

**Table 2:** Mean percentage electrolyte leakage values of *AtACBP6*-overexpressing *B. napus*-RC lines and wild-type plants subjected to freezing-with-frosting treatment at the vegetative, flowering, early podding to seed-setting stages

Plant Line /genotype(g)	Developmental Stage			
	Vegetative	Flowering	Early-podding	Seed-setting
109	19.98^c^	69.50^ab^	50.73^b^	45.06^b^
1	30.19^bc^	68.57^b^	56.07^b^	32.92^c^
81	20.00^c^	60.58^b^	59.51^b^	41.25^bc^
111	34.76^b^	69.57^ab^	n/a	n/a
WT	66.81^a^	81.09^a^	96.06^a^	69.08^a^

One-way ANOVA for genotype in black (a-c), values in each column followed by the same letter are not significantly different from each other, n/a = not assessed (n = 5 plants per genotype)

#### Recovery from freezing treatment

Gradual accumulation of green shoots and flowers occurred throughout the recovery period after freezing-without-frost stress.

#### Shoots

**Fig. 4:**
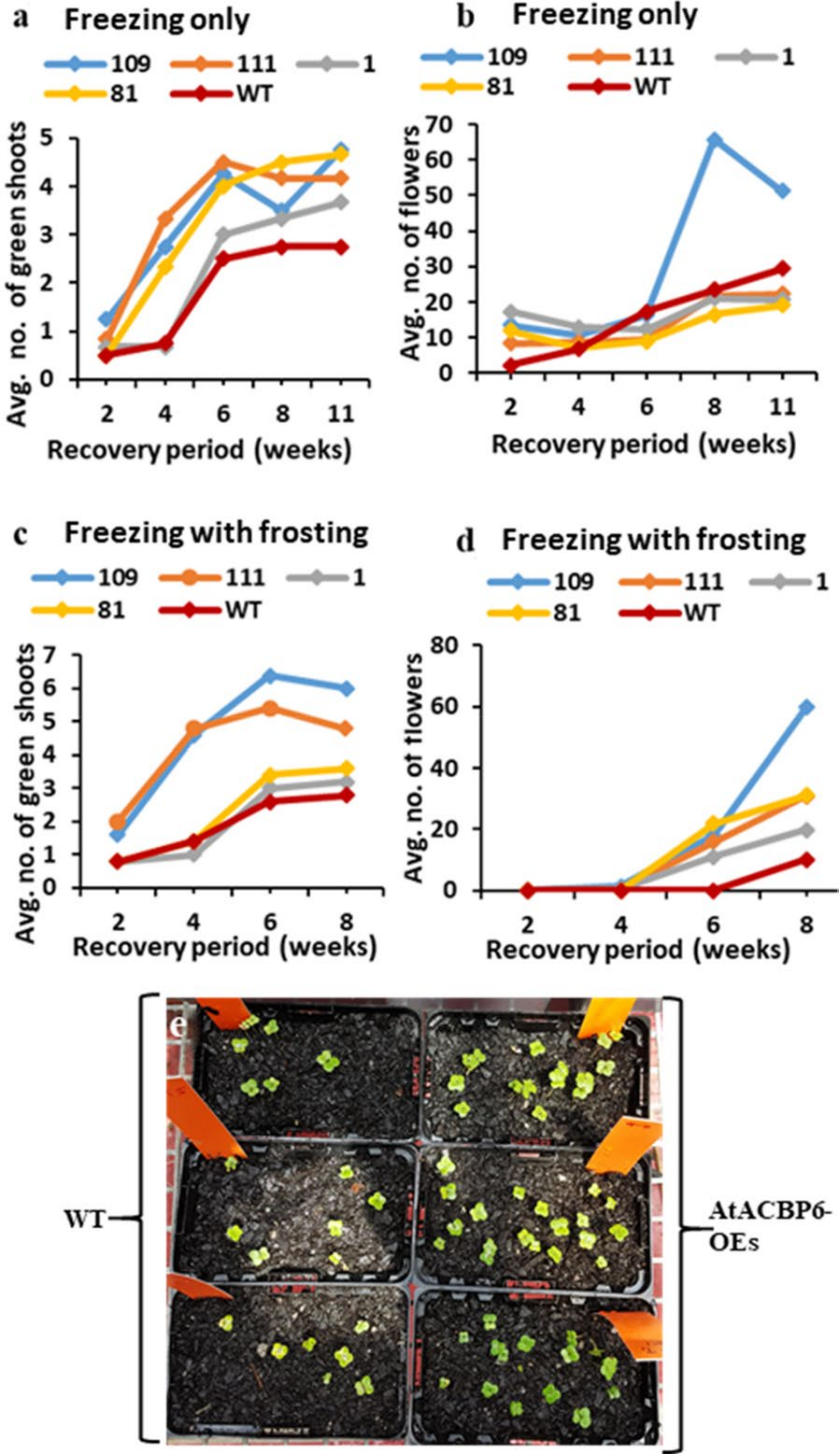
Recovery of *AtACBP6*-overexpressing *B. napus*-RC after freezing/frost treatment **a** Average number of shoots after freezing-without-frosting treatment. **b** Average number of flowers after freezing-without-frosting treatment. **c** Average number of shoots after freezing-with-frosting treatment. **d** Average number of flowers after freezing-with-frosting treatment during the recovery period. To provide statistical differences in shoot and flower numbers, the total area under the lines was calculated for each plant and the average values presented in Table 3. **e** Comparison of germination ability of wild type (left punnets) and AtACBP6-overexpressing seeds after freezing/frost treatment (right punnets) with *B. napus*-RC lines 81, 1 and 109 with 20 seeds/punnet

Wild-type plants did not produce as many new shoots as the *AtACBP6*-overexpressing lines (Fig. 4a). The average number of new shoots at harvest was 2.8 for wild type and between 3.7 and 4.8 for *AtACBP6*-overexpressing lines (Additional file 1: Table S4). However, perhaps owing to the small sample size, no significant difference was observed in the average number of new green shoots at each individual time interval (Additional file 1: Table S4). The number of new green shoots at four weeks was significantly higher (p < 0.05) for two transgenic *AtACBP6*-overexpressing lines, 109 and 111, than for *AtACBP6*-overexpressing lines 1, 81 and the wild-type lines (Additional file 1: Table S5). *AtACBP6*-overexpressing lines 111 and 109 performed best, producing around 5–6 new shoots by the end of the recovery period (Fig. 4c). To verify further the differences in shoot numbers, the area under the recovery curves (summarised in Fig. 4a, c) was calculated for each plant and average values presented in Table 3. The results indicate that freezing-treated *AtACBP6*-overexpressing lines 109, 111 and 81 recovered significantly better than the wild type, with respect to the production of new shoots.

**Table 3:** Average area under recovery curve calculated using the trapezoidal rule in Excel (see Fig. 4) for the number of green shoots and the number of flowers on *B. napus*-RC plants after freezing-without-frosting and freezing-with-frosting treatments

Plant Line/ genotype	Treatment	
	Freezing only	Freezing with Frost
*Shoots/Week: Average area under the curve over 11 weeks*		
109	31.1^a^	29.6^a^
111	30.6^a^	27.2^ab^
1	21.8^ab^	12.0^c^
81	29.9^a^	14.0^bc^
WT	15.8^b^	11.6^c^
*Flowers/Week: Average area under the curve over 11 weeks*		
109	215.8^a^	88.6^a^
111	119.0^bc^	58.4^b^
1	131.8^b^	40.2^bc^
81	100.5^bc^	70.0^ab^
WT	92.5^c^	10.2^c^

One-way ANOVA for genotype for each column (a-c), values followed by the same letter are not significantly different from each other (n = 5 plants per genotype)

#### Flowers

The average number of siliques and flowers at the 8-week-old stage was significantly higher (p < 0.05) for *AtACBP6*-overexpressing line 109 (65.8) than for any other line, while the lowest mean number of fertilised siliques and flowers was observed in the *AtACBP6*-overexpressing line 81 (16.5) (Additional file 1: Table S4). Two of the four transgenic lines were not protected from cold at the flowering stage but three of the four did gain an advantage from the presence of the *AtACBP6* cDNA when frost was present with a mean number of flowers at 8 weeks was 10 for wild type versus 19–60 for the transgenic lines (Additional file 1: Table S5).

Flower production was also relatively poor for the wild-type plants (10 to 15 flowers per plant), while *AtACBP6*-overexpressing lines were able to produce approximately 30 flowers on an average (Fig. 4d). Flower numbers recorded at 8-weeks showed significant variation among plant lines, with the wild type and *AtACBP6*-overexpressing line 1 generating significantly fewer flowers compared to the other lines (81, 109,111) (Additional file 1: Table S5). In the wild type, flowers did not appear by 6-weeks after frost-treatment while all *AtACBP6*-overexpressing lines had flowers by 6 weeks after treatment. *AtACBP6*-overexpressing line 109 produced a strikingly higher number of flowers (~ 60) than other lines (Additional file 1: Table S5).

Apart from *AtACBP6*-overexpressing line 1, frost-treated transgenic lines showed significantly higher production of flowers over 8-weeks in comparison to the wild type (Fig. 4b, d and Table 3).

#### Seed weight and germination percentages

The lowest dry pod/seed weights were displayed by the wild type and the *AtACBP6*-overexpressing line 81. *AtACBP6*-overexpressing lines 109, 111 and 1 showed harvest indexes (the ratio of harvested grain yield to total biological yield) of 0.20 or more, while the values of wild type and *AtACBP6*-overexpressing line 81 were approximately 0.10 (Table 4). Seeds collected from freezing-with-frost treated seed-setting *AtACBP6*-overexpressing lines 109 and 1 showed significantly higher germination percentages (at 80% and 76.5%, respectively) when compared to the wild type (31.3%), although the results from transgenic line 81 (germination 18.9%) were not significantly different from wild type (Table 5). There weren’t enough mature pods in line 111 to test the germination percentage.

**Table 4:** Harvest index and other yield parameters of harvested *B. napus*-RC plants subjected to freezing treatment without frosting

Genotype (g)	Total pod weight (dry) in g	Dry seed weight in g	Harvest Index
109	0.62^a^	0.41^a^	0.20^ab^
111	0.49^ab^	0.28^ab^	0.20^ab^
1	0.43^ab^	0.30^ab^	0.25^a^
81	0.26^b^	0.13^b^	0.11^b^
WT	0.29^b^	0.15^b^	0.09^b^

One-way ANOVA for genotype for each column (a–c), values in the same column followed by the same letter are not significantly different from each other (n = 5 plants per genotype)

**Table 5:** Viability of *B. napus*-RC seeds collected from early-podding to seed-setting plants after freezing treatment with frosting, as seen by seed germination

Plant line/genotype (g)	Seed germination percentage
109	80.0^a^
1	76.5^a^
81	18.8^b^
WT	31.3^b^

One-way ANOVA for genotype (a-b). Values in the same column followed by the same letter are not significantly different from one another (n = 4 plants per genotype)

## Discussion

### Transformation system

A genetic transformation system was developed for *B. napus*-RC plants by optimising the explant type, PGRs and the parameters of *Agrobacterium*-mediated transformation system. The optimised transformation system was used to generate *AtACBP*-overexpressing *B. napus*-RC, which showed enhanced cold tolerance.

Cotyledon explants of *B. napus*-RC grown in MS media supplemented with 1-naphthalene acetic acid (NAA) at 0.1 mg/L and 6-benzylaminopurine (BAP) at 1.0 mg/L, 0.01 mg/L gibberellic acid (GA3) and 5 mg/L silver nitrate produced a plantlet regeneration efficiency of 70%, which was higher than the regeneration efficiency from hypocotyls. Thus, cotyledons represent the best explant type for transformation experiments with rapid-cycling lines. Kamal et al*.* [41] also reported higher regeneration frequencies for cotyledons (31–100%) than hypocotyls (6–44%) in three commercial canola cultivars (Surigol®, Quantum®, Option 500®). In contrast, Maheshwari et al*.* [22] found that plants of the *B. napus* cultivar Invigor 5020® displayed greater (~ 20% more) shoot induction with hypocotyls than with cotyledons and shoot induction was faster in hypocotyls than cotyledons. These results suggest that the explant response in tissue culture media is likely to be genotype dependent.

#### Shoot production

The addition of AgNO_3_ to the medium was essential for obtaining a high-level shoot induction from callus cells. Silver ions have been shown to act as ethylene antagonists by replacing copper ions in the ethylene receptor, which stops activation of the receptors and, thus, prevents ethylene responses [42, 43]. Shoot induction was significantly reduced in the absence of AgNO_3_ in the medium, with initiated shoots appearing stunted, suggesting the importance of AgNO_3_ for healthy shoot production from *B. napus* explants. However, in contrast, Teo et al*.* [44] reported a significant decrease in shoot induction from rapid-cycling *B. rapa*, when AgNO_3_ was added to the SIM, and instead used the ethylene inhibitor aminoethoxyvinylglycine (AVG) to improve shoot induction.

The average proportion of explants producing shoots was 70% on SIM supplemented with 1 mg/L BAP, 0.1 mg/L NAA,5 mg/L AgNO_3_ and 0.01 mg/L GA_3_. A comparatively low shoot induction rate of 33% was reported from rapid-cycling *B. rapa* by Teo et al*.* [44] with 4.5 mg/L BAP, 0.37 mg/L NAA and 0.3 mg/L AVG in the SIM. In a similar experiment, Cogbill et al*.* [19] achieved a regeneration percentage of 44.5% from 4-day-old cotyledons of rapid-cycling *B. rapa*, using media supplemented with thiadiazuron (1.5 mg/L) and NAA (0.5 mg/L). These authors also reported that regeneration declined to 32.5% for 5-day-old explants and 23% for 9-day-old plants, suggesting that the use of young explants is crucial in tissue culture regeneration.

#### Root production

Rooting of tissue culture regenerated shoots is a critical step in plant regeneration and IBA is an essential supplement in the RIM. 1–2 mg/L of IBA delivered a higher rooting ability than 0.5 mg/L or less in the medium. To avoid the development of roots directly from the callus tissue, removing the callus cells from the crown area was important before transferring the shoots into the RIM. This prevented rooting from callus tissue and encouraged the development of proper vascular connections with the stem. Washing the roots thoroughly to remove any traces of MS medium before transferring in vitro plants into potting soil was also important to minimise colonisation by potentially pathogenic soil bacteria and fungi. A similar procedure was described by Conner and Thomas [45] for establishing plants that are amenable to tissue culture regeneration.

Four-day-old cotyledon explants co-cultivated in *Agrobacterium* inoculum at 0.1 optical density at 600 nm (approximately 0.1 × 109 cfu/mL) showed an average transformation efficiency of 16.4% when cotyledon explants were sectioned in the absence of liquid medium, dipped for 30–60 s in the *Agrobacterium* inoculum and exposed to the initial antibiotic selection medium (25 mg/L kanamycin) two weeks after co-cultivation. The first transformation experiments following the protocol of Boszoradova et al*.* [25] and Liu et al*.* [8] produced a transformation efficiency of between 2 and 5%. However, delaying the first round of antibiotic selection (25 mg/L kanamycin) by a week and modifying the explant preparation method resulted in an improved efficiency for the rapid-cycling plants. The use of a dry surface to prepare the explants and immediate dipping of the explant in the *Agrobacterium* solution most likely allowed more contact of *Agrobacterium* cells with the cut surface of the cotyledon, increasing the probability of obtaining transformed callus cells. In addition, the delay in commencing antibiotic selection by an additional week presumably provided adequate time for the transformed callus cells to establish and proliferate before being subjected to the antibiotic selection pressure. Possible escapees were eliminated using the two rounds of antibiotic selection (25 mg/L and 50 mg/L kanamycin) and the shoots that survived selection were confirmed as transgenic by PCR. Similarly, Bhuiyan et al*.* [46] observed a 14% rise in transformation efficiency of *B. juncea* by delaying the initial antibiotic selection of cotyledon explants.

#### Frost tolerance trait trial

Frost tolerance was selected for trait testing because of its potential importance to the Australian canola industry [35]. Chen et al*.* [36] and Liao et al*.* [37] established the potential for enhanced cold tolerance when *AtACBP6* is overexpressed in *Arabidopsis.* Based on these observations, *B. napus*-RC lines overexpressing *AtACBP6* were generated and subsequent testing of the resulting transgenic lines under low temperature stress at the vegetative to seed-setting stages revealed reduced electrolytic leakage indicating lowered membrane damage. Frost-treated vegetative stage *AtACBP6-*overexpressing *B. napus*-RC plants showed less than half the electrolyte leakage (20%-30%) compared to wild-type plants (67%). Megha et al*.* [47] using a 3-week-old spring variety of *B. napus*, observed a gradual increase in electrolyte leakage during a 4 °C incubation period, with a maximum value of approximately 11% being reached after a 48-h exposure. This electrolyte leakage value is significantly lower than the values observed in wild-type *B. napus-*RC subjected to freezing stress.

At the flowering to early podding stage, the percent electrolyte leakages were between 51 and 70% in *AtACBP6-*overexpressing *B. napus*-RC plants, while they were between 81 and 96% in wild-type rapid-cycling plants. In both the cases higher electrolyte leakage was observed compared to pre-flowering plants.

Overall, the electrolyte leakage results suggest that the over-expression of the *AtACBP6* transgene significantly reduced cell membrane damage, with improved membrane integrity [40] retained during low temperature stress across the four different development stages.

After exposure to cold/ freezing conditions, *AtACBP6*-overexpressing lines started to produce new shoots and flowers more rapidly than the wild-type plants. Cold stressed transgenic *B. napus*-RC plants retained a harvest index of around 0.20 in the glasshouse, close to the average for unstressed commercial canola under field conditions (between 0.2 and 0.3) [48, 49].

Mature seeds (~ 20–25 days after pollination) obtained from the *AtACBP6*-overexpressing lines 109 and 01 showed 70–80% germination, confirming that their embryos are significantly less affected than those of the wild type. In a similar study, Lardon and Triboi-Blondel [50] subjected winter rapeseed (*Brassica napus*, cv Samourai) plants to a − 3 °C treatment for 4 h and reported that the sensitivity of ovules to freezing increased from 8-days before anthesis to anthesis. Interestingly, they found that pollen viability was not affected if the stress was applied after the binucleate stage. It was also found that seeds were increasingly susceptible from just after fertilisation to 20-days after fertilisation whereas freezing stress applied on seeds 35-days after fertilisation had much less effect. Lardon and Triboi-Blondel [50] suggested that during the late embryonic phase, increased production of late embryogenesis-abundant (LEA) [51] mRNAs assists in lowering the freezing sensitivity of seeds.

## Conclusion

This study reports a transformation protocol for *B. napus*-RC lines and has applied it to the generation of *AtACBP6*-overexpressing rapid-cycling transgenic plants. Four months were required to generate T_o_ *B. napus*-RC lines compared to more than six months for establishing transgenic Westar *B. napus*. Conventional canola varieties also require many times the growing space compared to the *B. napus*-RC with resulting implications for the costs of experiments/trials. Thus, *B. napus*-RC has the potential to be used as a trait testing platform for canola. The freezing/frost assays described suggests that the *AtABCP*-overexpressing *B. napus*-RC are more tolerant to cold stress than the wild type. However, this is a preliminary investigation and larger scale studies with more replications will be required to further confirm the role of ACBP6 in frost tolerance in *B. napus*. The introduction of *AtACBP6* into commercial *B. napus* cultivars may have the potential to reduce the crop damage that occurs during moderate to severe frost events in oilseed growing regions in Australia and in northern Europe/America. Given the similarity of *B. napus* acyl-CoA-binding proteins (BnaA05g36060D, BnaA08g07670D, BnaAnng25690D, BnaCnng15340D) to *Arabidopsis* acyl-CoA-binding proteins (82.6% average amino acid sequence identity) [52], it might be possible to utilise genome editing techniques such as the CRISPR/Cas9 system to perform targeted gene editing of *B. napus ACBP* promoters to elevate expression of these genes. Plants with edited native genomes, rather than carrying transgenes, require less onerous safety evaluations and thus, may be more acceptable to industry for economic reasons and to regulators and consumers.

## Methods

### Plant materials and growth conditions:

Rapid-cycling *B. napus* seeds (seed stock CrGC 5–1), originally obtained from the rapid-cycling *Brassica* collection of the University of Wisconsin, Madison [10]. Seeds were sown in Debco® potting mix (50% composted medium bark, 40% composted coarse bark, 10% sand, with addition of Saturaid™, lime, iron, nitrogen and trace elements) and maintained in the glasshouse at 23–25 °C under 16 h light supplied by GE Lucalox™ PSL (GE Lighting, USA). For regeneration and transformation experiments, *B. napus*-RC seeds were surface sterilised and sown aseptically on half-strength MS medium [39].

### Assessment of shoot regeneration from cotyledons and hypocotyls in *B. napus*-RC

The aseptic preparation of cotyledon explants from four-day-old in vitro grown plants and the callus induction medium (CIM), shoot induction medium (SIM) and shoot elongation medium (SEM) were initially prepared following the methods of Boszoradova et al*.* [25], Maheshwari et al*.* [22] and Cardoza and Stewart [28]. Seven-day-old in vitro grown plants were used to prepare hypocotyl explants according to Cardoza and Stewart [28]. The explants were cultivated on MS medium in Petri plates with a range of PGR concentrations, 1-naphthaleneacetic acid (NAA) and 6-benzylaminopurine (BAP) (Additional file 1: Table S1) for callus induction. AgNO_3_ at 5 mg/L and gibberellic acid (GA_3_) at 0.01 mg/L were added to each treatment. Petri dishes were covered with aluminium foil and kept at 24 °C in an environmental chamber (Panasonic MLR-352-PE Climate Chamber, Panasonic Healthcare Co., Ltd. Japan) for 1-week for callus induction. The explants were then transferred to fresh media for shoot induction. Petri plates were kept under long days (16 h L/8 h D) at 24 °C in an environmental chamber at 60% relative humidity with a photosynthetic photon flux density of ~ 100 μmol·m^−2^·s^−1^ for shoot induction. After 4 weeks, the number of individual cotyledons producing green and healthy shoots in the SIM was counted.

### Optimisation of silver nitrate (AgNO_3_) levels for shoot regeneration from cotyledon explants

Explants prepared from 4-day-old cotyledons were grown in CIM and SIM containing NAA (0.1 mg/L), BAP (1.0 mg/L) and GA_3_ (0.01 mg/L) with treatments of AgNO_3_ (0, 2.5, 5.0 and 7.5 mg/L). After a week, explants with callus cells were transferred to SIM for a further 2 weeks. The number of individual cotyledons producing green and healthy shoots in the SIM were recorded.

### Optimisation of plant growth regulators for callus induction, shoot induction, shoot elongation, and root induction

Callus induction from cotyledons was tested using six different treatments (Additional file 1: Table S6). The number of explants producing calli were counted after one week of incubation in the CIM. Shoot induction was assessed in 7 different treatments (Additional file 1: Table S7). The number of explants producing shoots was recorded after 2 weeks in the SIM. The effect of six concentrations of BAP from 0 to 1 mg/L (Additional file 1: Table S8) on shoot length was assessed in the SEM after 2 weeks of growth in the media. Root induction from well-developed plantlets was evaluated at four concentrations of IBA in the RIM (Additional file 1: Table S9). The number of plantlets in the medium which had five or more well-developed lateral roots was counted at 3 weeks.

### Optimisation of *Agrobacterium*-mediated transformation of *B. napus*-RC

Plasmid pAT593 (CaMV *35S::AtACBP6*; Additional file 1: Fig. S3) was constructed by placing the *AtACBP6* cDNA contained within a *Bam*HI-*Sal*I fragment obtained from pAT332 [36] into identical sites located in vector pSa13 [53]. Two different *Agrobacterium*-mediated transformation procedures for rapid-cycling plants were assessed. The first, was based on the methods of Boszoradova et al*.* [25] and Liu et al*.* [8] with modifications. A single isolated *Agrobacterium* colony bearing the pAT593 plasmid (*AtACBP6* under the control of the CaMV *35S* promoter with a *NOS* terminator plus *NPTII* for kanamycin resistance, driven by the *NOS* promoter and *NOS* terminator) was grown for 36 h in Luria–Bertani (LB) liquid medium containing 50 mg/L kanamycin and 50 mg/L gentamycin at 28 °C, in a shaker incubator at 220 rpm.

Bacterial cells were pelleted at 4,400 rpm for 10 min before resuspending in MS liquid medium. Optical density (OD_600_) [8, 25] quantified concentration of bacteria per mL of liquid and adjusted to an OD_600_ of 0.10, 0.20 and 0.25, respectively. Aseptically prepared four-day-old cotyledon explants of *B. napus*-RC were dipped in these different *Agrobacterium* solutions and co-cultivated in the dark, in CIM with NAA (0.1 mg/L), BAP (1.0 mg/L), AgNO_3_ (5 mg/L) and GA_3_ (0.01 mg/L) for 2 days. After 48 h of co-cultivation, the explants were transferred to CIM which also contained the antibiotic carbenicillin (500 mg/L) to inhibit excessive *Agrobacterium* growth around the explants. After 1-week in the dark in CIM, explants were transferred into SIM which was supplemented with NAA (0.1 mg/L), BAP (1.0 mg/L), AgNO_3_ (5 mg/L) and GA_3_ (0.01 mg/L), carbenicillin (500 mg/L) plus the antibiotic kanamycin (25 mg/L) and kept in 16 h light at a photosynthetic photon flux density of ~ 100 μmol·m^−2^·s^−1^ at 60% humidity for transgenic shoot selection. After 2–4 weeks, the fully-grown shoots were aseptically separated from callus tissue using a sharp scalpel blade and transferred into SEM containing BAP (0.00125 mg/L) plus kanamycin (25 mg/L). After 2 weeks in SEM, the green shoots were transferred into medium with a higher selection pressure of 50 mg/L kanamycin.

The second transformation procedure involved modifications to the bacterial culture, explant preparation technique and application of antibiotic selection. *Agrobacterium* colonies containing plasmid pAT593 were grown for 16–24 h [28] to an OD_600_ = 1.0 at 600 nm. Bacterial cells were pelleted and resuspended in MS liquid and adjusted to OD_600_ = 0.10. The explant preparation method was modified by eliminating the MS liquid from the preparation medium. Once prepared, the cut ends were immediately dipped in *Agrobacterium* solution for 30 s and co-cultivated. A selection pressure of 25 mg/L kanamycin was applied in the SIM two weeks after co-cultivation with *Agrobacterium,* a delay of one week [25].

### Generation of transgenic *B. napus* rapid-cycling and Westar* AtACBP6*-overexpressing lines

Aseptically prepared four-day-old cotyledon *B. napus*-RC explants were co-cultivated with *Agrobacterium* cells harbouring the pAT593 plasmid as detailed above at a bacterial optical density of 0.10. To avoid using genetically identical shoots, only one healthy shoot was extracted per explant at the shoot induction stage. After 2 weeks in SEM, the green shoots were transferred into medium with a higher selection pressure of 50 mg/L kanamycin. Putatively transformed shoot initials which survived both rounds of selection and remained green were transferred into root induction medium supplemented with 2 mg/L IBA. Once the shoots had developed several healthy roots, they were carefully removed from the rooting medium and washed with lukewarm tap water to remove traces of gel. The plants were then transferred into Jiffy pots (Jiffy-7® peat pellets) under in vitro conditions to further enhance root induction. After the roots were fully established, these potentially transgenic plants were transplanted into pots with Debco® potting mix (50% composted medium bark, 40% composted coarse bark, 10% sand, Saturaid™, lime, iron, nitrogen and trace elements) and maintained in the glasshouse at 23–25 °C under 16 h light supplied by GE Lucalox™ PSL (GE Lighting, U.S.A). They were fertilised initially with slow-release fertiliser ‘Osmocote®’ granules and bi-weekly with ‘Aquasol®’ fertiliser as directed by the manufacturer. Once these plants were well established in the glasshouse, they were characterised using molecular techniques. *AtACBP6-*overexpressing *B. napus* cv. Westar plants were generated using the same protocol as above.

### Molecular characterisation of transgenic plants overexpressing *AtACBP6*

For screening of putatively transformed shoots, leaf samples were collected from young green shoots grown in the SEM supplemented with kanamycin. DNA was extracted using a published crude extraction method [54]. A total reaction volume of 20 µL (× 1 PCR buffer, 0.2 mM dNTPs (Bioline), 0.2 µM of each primer (Sigma) (Additional file 1: Table S10), 2 mM MgCl_2_ (Bioline), 1-unit Mango™ Taq polymerase (Bioline) and 40 ng of gDNA) was used in each PCR reaction. PCR products were separated by gel electrophoresis and visualised.

RNA was extracted from putatively transformed plants using TRIzol reagent (Invitrogen) according to the manufacturer’s instructions. RNA was treated with DNase (TURBO DNA-free kit Invitrogen) to remove any traces of genomic DNA. One µg RNA was reverse transcribed using iScript™ cDNA synthesis kits (Bio-Rad) according to the manufacturer’s instructions. A 1.5 µL aliquot from each cDNA sample was used in conventional PCR with gene-specific primers.

### Western blot analysis on *AtACBP6*-overexpressing *B. napus*

Western blot analysis of PCR-confirmed transgenic *B. napus*-RC and Westar lines was performed on total plant protein extracted from 3-week-old *B. napus* wild-type plants and *AtACBP6* plants following the method of Shewey and Fido [55]. The concentration of the protein samples was measured using the Micro Lowry method with Peterson’s modification (Sigma) as per the manufacturer’s instructions. Forty-five µg protein was loaded per sample for SDS-PAGE. Proteins were transferred to Amersham Hybond-P Polyvinylidene Fluoride (PVDF) membrane and the immunoblotting was performed with anti-AtACBP6 [36] antibodies to confirm protein expression in transgenic lines.

### Phenotypic characterisation of *B. napus*-RC* AtACBP6* lines

Transgenic *B. napus*-RC *AtACBP6*-overexpressing lines and wild-type plants at the vegetative stage were tested for their freezing/frost tolerance ability according to a vegetative stage plant screening protocol by Fiebelkorn and Rahman [5] with minor modifications (Additional file 1: Fig. S5). Freezing without frosting treatment on the vegetative plants started at 4 °C and dropped in steps to approximately − 3 °C at a rate of 2 °C/h. Plants were kept at −3 °C (−2.9 ± 0.5 °C recorded by the temperature logger located inside the cabinet) for 1 h and the temperature was then increased back to 4 °C at the same rate. The next oldest leaf below the youngest fully expanded leaf was collected to take electrolyte leakage measurements. The plants were allowed to recover at 4 °C for 24 h and then moved back into the glasshouse. Freezing with frosting treatment on the vegetative plants started at 4 °C and was dropped to 0 °C at a rate of −2 °C/h. Ice crystals were placed on the soil surface in each pot to induce an artificial frosting event. The temperature was further decreased to −3 °C. After 1 h at −3 °C (−2.6 ± 0.4 °C recorded in the logger), the temperature was gradually increased to 4 °C at the same rate and the next oldest leaf below the youngest fully expanded leaf was collected to take electrolyte leakage measurements. Plants were then allowed to recover at 4 °C for 24 h before moving them to the glasshouse (Additional file 1: Fig. S5).

Frost treatments at the flowering stage and the seed-setting stage were based on Chen et al. [56]. In the freezing with frosting treatment of the flowering, early-podding and seed-setting plants, the temperature was dropped from 4° C to −2° C at a rate of 1 °C/ h. Once −2 °C was reached, ice crystals were introduced on to the soil to induce frost. Plants were kept at −2 °C for 2 h and the temperature was then lowered at 1 °C/h to −4 °C. After an hour, the temperature was increased over one hour back to 4 °C, at which temperature plants thawed and were allowed to recover for 24 h before being moved back to the glass house. The leaf below the youngest fully expanded leaf was collected to take electrolyte leakage measurement once the temperature inside the cabinet reached 4 °C (Additional file 1: Fig. S5).

### Recovery of freezing-without-frost-treated and freezing-with-frost treated plants

Plants were monitored every two weeks following freezing/frost treatments, to record new shoot and bud formation, and flowering and silique formation according to Gusta [57] and Gusta and Wisinewski [58]. Five plants per genotype per treatment were evaluated. Plants were evaluated every two weeks.

The fresh total plant biomass of the freezing-without-frost treated *B. napus*-RC was measured at harvest after 11 weeks. Then the plants were oven dried at 70 °C for one week, by which time a constant dry weight was obtained. The dry weights of shoots, roots and seeds of these plants were measured and were used to calculate the harvest index according to Schauer et al*.* [59]. Freezing-with-frost treated *B. napus*-RC were maintained until the flowering to early seed-setting stage with the number of flowers closely monitored.

### Electrolyte leakage measurements of leaves

Plant tissue sampling was based on the protocol by Rapacz [60]. The measurements were taken immediately after the end of the freezing stress on wild-type and transgenic leaves using a benchtop conductivity meter, Oakton CON 700, following Prasil and Zamecnk [61] and Rapacz (60). For each plant, two leaf discs were prepared from the next oldest leaf below the youngest fully expanded leaf, using a 15 mm cork-borer and placed separately in 50 mL Falcon tubes having 15 mL of milli-Q water. Tubes were shaken at 80 rpm for 30 min before taking the initial electrolyte leakage measurements. The leaf samples were autoclaved at 121 °C for 15 min at 10.34 kPa to obtain the total remaining electrolyte content. Initial electrolyte leakage was expressed as a percentage of the total ionic strength (electrolyte leaked plus electrolyte remaining).

### Seed analysis using seed germination

Mature seeds from frost-treated, seed-setting plants were collected and sown in Debco® potting mix (50% composted medium bark, 40% composted coarse bark, 10% sand, Saturaid™, lime, iron, nitrogen and trace elements) and maintained in the glasshouse at 23 °C under 16 h light. After 8-days, the proportion of successfully germinated ≥ 3 cm tall seedlings was calculated as a percentage of the total seed number which had been sown.

### Statistical analysis

Data was analysed using Minitab 17 statistical analysis software. One-way ANOVA and Two-way ANOVA were used, and the means were compared using Fisher’s Least Significant Difference (protected) test at the 5% confidence interval. Paired T-tests were used to compare the measurements taken before and after freezing treatment.

## Supplementary Information

**Additional file 1.** Additional figures and tables.

## Data Availability

The data sets used during the current study are available from the corresponding author on reasonable request.
