# Supplementary material for: Overexpressing Arabidopsis thaliana ACBP6 in transgenic rapid-cycling Brassica napus confers cold tolerance
Source: Plant Methods. 2022 May 12;18:62. doi: 10.1186/s13007-022-00886-y (PMC9097446; doi:10.1186/s13007-022-00886-y)
Supplement: Supplementary file 1 — Additional file 1. Additional figures and tables. [file 13007_2022_886_MOESM1_ESM.docx]

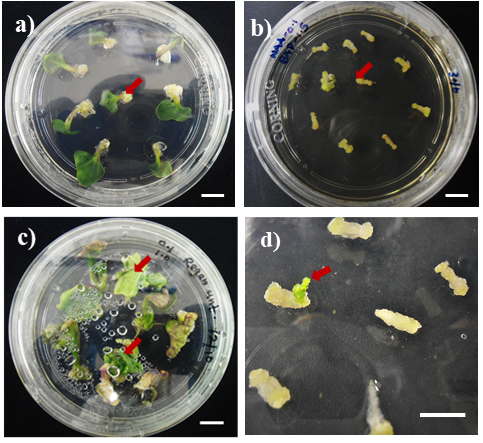


**Fig. S1:** Callus and shoot induction of *B. napus*-RC cotyledons and hypocotyls in tissue culture media supplemented with NAA (0.1 mg/L), BAP (1.0 mg/L), GA_3_ (0.01 mg/L) and AgNO_3_ (5 mg/L). **a)** Cotyledons with calli (arrow) at the petiole end are observed following 1-week in callus induction medium. **b)** Hypocotyls with calli (arrow) at the two cut ends 2 weeks in callus induction medium. **c)** Shoot-producing cotyledons 2-weeks in shoot induction medium. Arrows indicate shoot initials from cotyledon. **d)** Shoot-producing hypocotyls following 4 weeks on shoot induction medium. Red arrow indicates a shoot initial from hypocotyl. Scale bar, 1 cm.


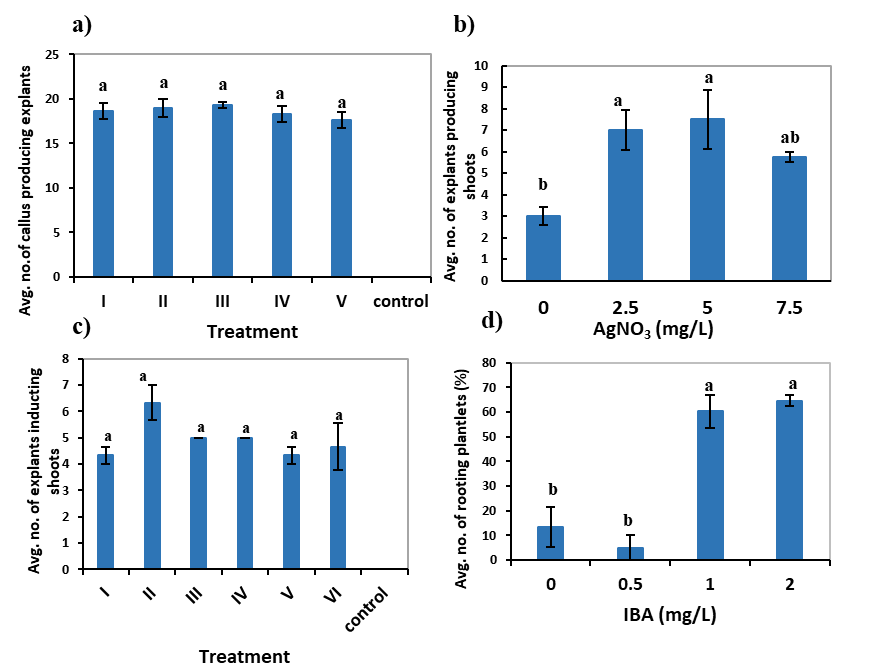


**Fig. S2:** Optimisation of plant growth regulators (NAA, BAP, GA_3_ and zeatin) and silver nitrate (AgNO_3_) and in *in vitro* regenration media of *B. napus*-RC **a)** The average number of explants producing calli in six CIM (see Table S6 for details) (n=20) **b)** the average number of explants producing shoots at 0, 2.5, 5.0 and 7.5 mg/L silver nitrate (n=10) **c)** The average number of explants producing shoots in seven SIM (see Table S7 for details) (n=9) **d)** Average number of shoots producing roots at 0, 0.5, 1.0 and 2.0 mg/L IBA (n=3 to 5). Error bars are standard error of the mean. Means that do not share the same letter are significantly different from each other according to Fisher’s Least Significance Difference (protected) test at the 5% confidence level.


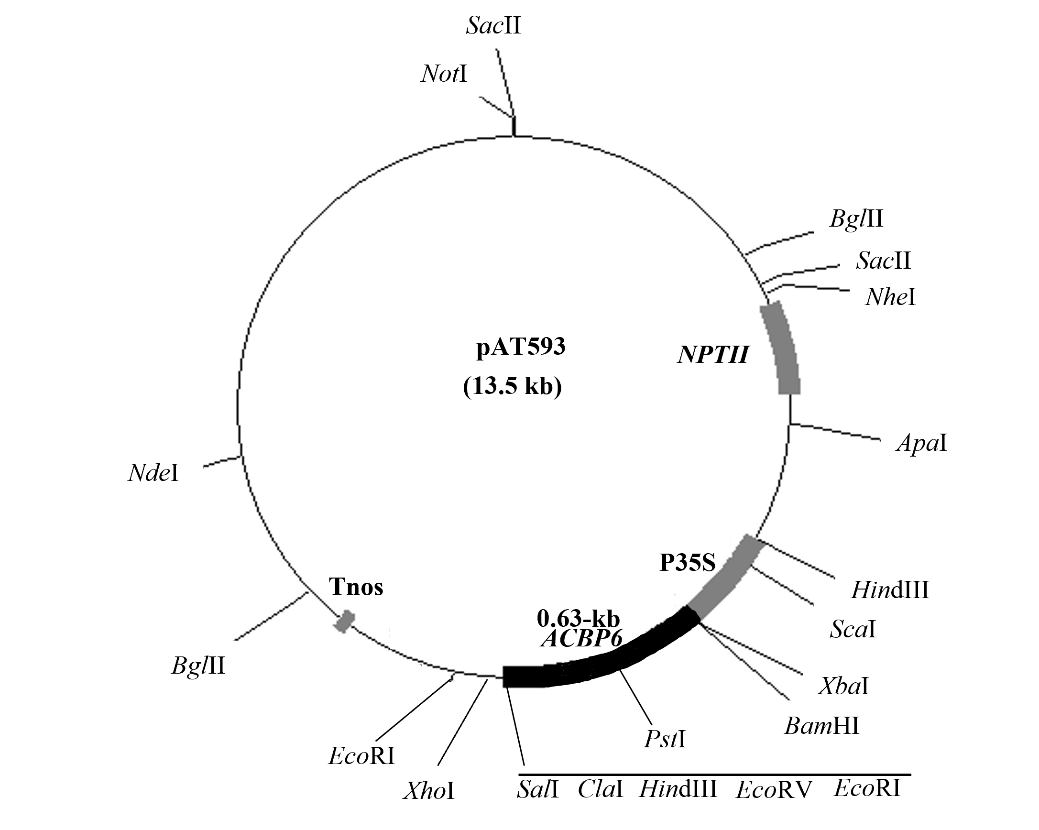


**Fig. S3:** Map of plasmid pAT593. This 13.5-kb plasmid was constructed to express *Arabidopsis* *AtACBP6* (AT1G31812) from the Cauliflower Mosaic Virus (CaMV) *35S* promoter (P35S). *NPTII* (gene encoding neomycin phosphotransferase II) confers resistance to kanamycin. Tnos is the transcription terminator of the gene encoding nopaline synthase (*NOS*). Restriction enzyme sites are also provided.


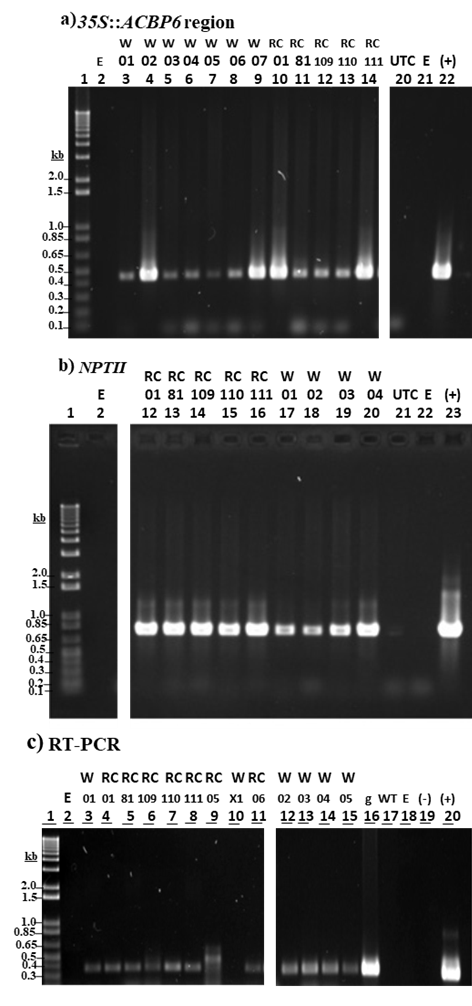


**Fig. S4:** PCR confirmation of independent *AtACBP6*-overexpressing *B. napus*-RC and *B. napus* Westar lines established in the glasshouse, using PCR and reverse transcription PCR. **a**) PCR amplification of the CaMV 35S::*ACBP6* region using primer pairs 35SB/ML838 (amplicon size 0.4kb). Lane 1, 1 kb Plus DNA ladder; Lane 2, empty; lanes 3-9, *B. napus* Westar (W) *AtACBP6*-overexpressing lines 01, 02, 03, 04, 05, 06, and 07, respectively; Lanes 10 to 14, *B. napus*-RC (RC) *AtACBP6*-expressing lines 01, 81, 109, 110 and 111, respectively; Lane 20, untransformed control (UTC); Lane 21, empty (E); Lane 22, plasmid pAT593 control (+). **b)** PCR amplification of *NPTII* transgene (0.7 kb) using primer pairs NPTII-2F/NPTII-2R (amplicon size, 0.7 kb). In b) Lanes 12-16, *B. napus-*RC lines 01, 81, 109, 110 and 111, respectively; Lanes 17-20, *B. napus* Westar lines 01, 02, 03 and 04, respectively; Lane 21, untransformed control; Lane 22, empty; Lane 23, plasmid pAT593 control. **c)** Reverse transcription PCR (RT-PCR) using ML750/ACBP02 primers, amplifying the 0.36 kb *AtACBP6* cDNA. Lane 1, 1 kb Plus DNA Ladder; Lane 2, empty, Lanes 3 to 11, cDNA samples of putative AtACBP6 *B. napus* where lanes 4-9 and 11 are *B. napus*-RC lines 01, 81, 109, 110, 111, 05 and 06. Lane 3 and 10 are *B. napus* Westar lines 01 and x1. Lanes 12-15 are *B. napus* Westar lines 02, 03, 04 and 05. Lane 16, *AtACBP6* genomic DNA PCR control (g); Lane 17, wild type *B. napus*; Lane 18, no template control (-); Lane 19, empty; Lane 20, plasmid pAT593 control (+).

**
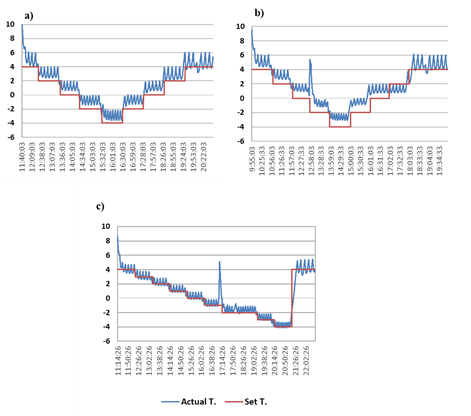
**

**Figure S5**: Three freezing/frost regimes used to screen freezing/frost tolerance ability of *AtACBP*6-overexpressing *B. napus*-RC plants. **a)** Freezing-without-frosting treatment applied on 4-week-old vegetative plants [min. T. = -2.9 ± 0.5^o^C], **b)** Freezing-with-frost treatment applied on 4-week-old vegetative plants [min. T. = -2.6 ± 0.4^o^C] **c)** Freezing-with-frost treatment applied on flowering to seed-setting plants where min. T. recorded for 9-week-old flowering plants = -3.7 ± 0.3^o^C, min. T. recorded for 10-week-old early-podding stage plants = -5.2°C ± 0.79 and min. T. recorded for 12-week-old seed-setting stage = -3.6 ± 0.5^o^C. The spikes in b) and c) reflect the times at which the door was opened to place ice crystals on the soil surface in each pot.

**Table S1**: The percentage of *B. napus*-RC explants inducing calli (after 2 weeks in CIM) and shoots (after 4 weeks in SIM) for treatments 1 to 9. The concentrations of 1-naphthaleneacetic acid (NAA) and 6-benzylaminopurine (BAP) are given in mg/L. Means in the same column that do not share the same letter are significantly different (p<0.05) from each other according to Fisher’s Least Significance Difference (protected) test (n=10, r=4).

| Treatment number | Concentration of NAA and in mg/L | Concentration BAP in mg/L | Mean percentage of explants producing callus cells | Mean percentage of explants producing shoots |
| --- | --- | --- | --- | --- |
| 1 | 0.1 | 0.5 | 93^a^ | 16^a^ |
| 2 | 0.1 | 1.0 | 84^ab^ | 26^a^ |
| 3 | 0.1 | 1.5 | 72^abc^ | 09^a^ |
| 4 | 0.2 | 0.5 | 72^abc^ | 09^a^ |
| 5 | 0.2 | 1.0 | 64^bc^ | 08^a^ |
| 6 | 0.2 | 1.5 | 49^c^ | 15^a^ |
| 7 | 0.3 | 0.5 | 80^ab^ | 09^a^ |
| 8 | 0.3 | 1.0 | 64^bc^ | 11^a^ |
| 9 | 0.3 | 1.5 | 51^c^ | 07^a^ |

**Table S2**: Percentage of putative *AtACBP6*-overexpressing *B. napus*-RC shoots from preliminary experiments using the pAT593 plasmid, which remained green in the kanamycin (25 mg/L) media and were later confirmed to be PCR-positive. *Agrobacterium* concentration is indicted by the optical density value at 600 nm (OD_600_), dipping time is displayed in sec, the number of green shoot-producing explants subsequently confirmed by PCR is expressed as a percentage of the total explants used in each experiment ± standard error of mean.

| OD_600_ | Dipping time  in sec | No. of cotyledons producing green shoots confirmed as transgenic by PCR | Total number of explants | Percentage of explants producing green shoots confirmed as transgenic by PCR | Average percentage of explants producing green shoots confirmed as transgenic by PCR ± SEM |
| --- | --- | --- | --- | --- | --- |
| 0.1 | 30 | 6 | 250 | 2.4 | 5.0 ± 0.75 |
|  |  | 2 | 60 | 3.3 |  |
|  |  | 2 | 29 | 6.8 |  |
|  |  | 4 | 60 | 6.6 |  |
|  |  | 4 | 63 | 6.3 |  |
|  |  | 3 | 63 | 4.7 |  |
| 0.2 | 30 | 1 | 50 | 2 | 2.0 ± 1.37 |
|  |  | 1 | 17 | 5.8 |  |
|  |  | 0 | 31 | 0 |  |
|  |  | 0 | 53 | 0 |  |
| 0.25 | 30 | 1 | 46 | 2.1 | 2.8 |
|  |  | 1 | 28 | 3.5 |  |
| 0.2 | 120 | 2 | 54 | 3.7 | 3.2 ± 1.3 |
|  |  | 0 | 27 | 0 |  |
|  |  | 1 | 40 | 2.5 |  |
|  |  | 2 | 19 | 10.5 |  |
|  |  | 1 | 36 | 2.7 |  |
|  |  | 1 | 36 | 2.7 |  |
|  |  | 0 | 25 | 0 |  |

**Table S3:** Observed transformation efficiencies of *B. napus*-RC cotyledons in three indepedent experiments using plasmid pAT593 and optimal transformation conditions (0.1 mg/L NAA, 1.0 mg/L BAP and 5.0 mg/L AgNO_3_). All experiments utilised the same *Agrobacterium* concentration (OD_600_=0.10) and dipping time (30 sec). MS liquid was not used in the explant preparation medium and 25 mg/l kanamycin selection was applied 2-weeks after co-cultivation. The percentage of explants giving rise to transgenic shoots was established by testing for the presence of the transgene in green shoots using PCR.

| Experiment | Total number of explants | Percentage of explants producing transgenic green shoots |
| --- | --- | --- |
| 1 | 61 | 16.4 |
| 2 | 45 | 13.3 |
| 3 | 107 | 19.6 |

**Table S4**: Recovery of *AtACBP6*-overexpressing lines and wild-type *B. napus*-RC plants after freezing-without-frosting treatment at the vegetative stage with respect to production of new shoots, flowers, siliques and pods. One-way ANOVA results per genotype in each column are indicated a-c. Values followed by the same letter are not significantly different, ***ns***= non significant T-value in one-way ANOVA (n=5 plants per genotype).

| *Genotype (g)* | *At 6 weeks* | | *At 8 weeks* | |
| --- | --- | --- | --- | --- |
|  | *Avg. number of shoots (ns)* | *Avg. number of flowers (ns)* | *Avg. number of shoots (ns)* | *Avg. number of flowers/siliques* |
| 109 | 4.3 | 16.3 | 3.5 | 65.8 ^a^ |
| 111 | 4.5 | 9.3 | 4.2 | 21.8 ^b^ |
| 1 | 3.0 | 12.3 | 3.3 | 21.0 ^b^ |
| 81 | 4.0 | 9.0 | 4.5 | 16.5 ^b^ |
| WT | 2.5 | 17.3 | 2.8 | 23.5 ^b^ |
| *Genotype (g)* | *At harvest (11 weeks)* | | | |
|  | *Avg. number of shoots (ns)* | *Avg. number of inflorescences* | *Avg. number of all siliques* | *Avg. number of pods with fully mature seeds* |
| 109 | 4.8 | 8.3^a^ | 51.3^a^ | 34.4^a^ |
| 111 | 4.2 | 6.5^ab^ | 22.3^bc^ | 16.3^b^ |
| 1 | 3.7 | 4.3^b^ | 20.7^bc^ | 18.6^b^ |
| 81 | 4.7 | 5.3^b^ | 19.2^c^ | 11.0^b^ |
| WT | 2.8 | 5.0^b^ | 29.5^b^ | 16.3^b^ |

**Table S5**: Recovery of *AtACBP6*-overexpressing lines and wild-type *B. napus*-RC plants after freezing-with-frosting treatment at the vegetative stage with respect to new shoots and flowers. One-way ANOVA results per genotype in each column are indicated a-c. Values followed by the same letter are not significantly different, ***ns***= no significant T-value in one-way ANOVA (n=5 plants per genotype).

|  | *At 4 weeks* | *At 6 weeks* | |
| --- | --- | --- | --- |
| *Genotype (g)* | *Avg. number of shoots* | *Avg. number of shoots (ns)* | *Avg. number of flowers* |
| 109 | 4.6^a^ | 6.4 | 18^ab^ |
| 111 | 4.8^a^ | 5.4 | 15.8^ab^ |
| 1 | 1.0^b^ | 3.0 | 11.0^b^ |
| 81 | 1.4^b^ | 3.4 | 21.8^a^ |
| WT | 1.4^b^ | 2.6 | 0.0^c^ |
| *Genotype (g)* | *At 8 weeks* | | |
|  | *Avg. number of shoots (ns)* | *Avg. number of flowers* | *Avg. number of inflorescences* |
| 109 | 6**.**0 | 59.8^a^ | 8.0^a^ |
| 111 | 4.8 | 30.8^b^ | 3.0^b^ |
| 1 | 3.2 | 19.8^bc^ | 2.2^b^ |
| 81 | 3.6 | 31.0^b^ | 2.2^b^ |
| WT | 2.8 | 10.2^c^ | 2.2^b^ |

**Table S6**: Composition of callus induction media (CIM) [I to VI] used to induce callus formation from *B. napus*-RC cotyledon explants. The concentration of each component is given in mg/L (n=20).

| Treatment | I | II | III | IV | V | VI |
| --- | --- | --- | --- | --- | --- | --- |
| AgNO_3_ | 5.0 | 2.5 | 5.0 | 5.0 | 5.0 | Ms only |
| NAA | 0.1 | 0.1 | 0.1 | 0.2 | 0.2 | 0.0 |
| BAP | 0.5 | 1.0 | 1.0 | 0.5 | 1.0 | 0.0 |

**Table S7**: Composition of shoot induction media (SIM) [I to VII] used to induce shoot formation from *B. napus*-RC from cotyledon explants. The concentration of each component is given in mg/L (n=10).

| Treatment | I | II | III | IV | V | VI | VII |
| --- | --- | --- | --- | --- | --- | --- | --- |
| AgNO_3_ | 2.5 | 5.0 | 2.5 | 5.0 | 2.5 | 5.0 | MS only |
| NAA | 0.1 | 0.1 | 0.1 | 0.1 | 0.0 | 0.0 | 0.0 |
| BAP | 1.0 | 1.0 | 5.0 | 5.0 | 4.0 | 4.0 | 0.0 |
| GA_3_ | 0.01 | 0.01 | 0.01 | 0.01 | 0.0 | 0.0 | 0.0 |
| Zeatin | 0.0 | 0.0 | 0.0 | 0.0 | 2.0 | 2.0 | 0.0 |

**Table S8**: Composition of shoot elongation media (SEM) [I-VI] used to induce shoot elongation in *B. napus*-RC cotyledon explants. The concentration of 6-benzylaminopurine (BAP) is given in mg/L (n=9).

| Treatment | I | II | III | IV | V | VI |
| --- | --- | --- | --- | --- | --- | --- |
| BAP | 0 | 0.00125 | 0.01 | 0.05 | 0.5 | 1 |

**Table S9**: Four root induction media (RIM) tested for root induction from *B. napus*-RC plantlets. The concentration of indole-3-butyric acid is given in mg/L (n=3 to 5).

| Treatment | I | II | III | IV |
| --- | --- | --- | --- | --- |
| IBA | 0 | 0.5 | 1.0 | 2.0 |

**Table S10**: Primer sequences and PCR conditions used for genotyping putative *AtACBP6*-overexpressing *B. napus*-RC shoots. The numbers indicate the position of the primer downstream of the transcription site.

| Primer pairs | Primer pair sequences (5’- 3’) and the position (kb) in the gene sequence | PCR conditions | Reference |
| --- | --- | --- | --- |
| 35SB F’  6ACBP02 R’ | CAATCCCACTATCCTTCGCAAGACC (25-49)  CAGGTTGAAGCCTTGGAAGCAGCA (360-383)  amplicon size: 0.42 kb | (95^o^C- 30 sec,  65^o^C- 30 sec,  72^o^C- 2 min)  37 cycles | (Chen et al., 2008) |
| ML838    ML750 | CAGGATCCTGAAGCCTTGGAAGCA  GCAACT (357-386)  ATATGGATCCCACGCGTTGTCCTCG  TCTTCT (19-49)  amplicon size: 3.7 kb | (94^o^C- 30 sec,  70^o^C- 45 sec,  72^o^C- 2 min)  37 cycles | (Chen et al., 2008) |
| 35SB F’  ML838 | CAATCCCACTATCCTTCGCAAGACC  (25-49)  CAGGATCCTGAAGCCTTGGAAGCA  GCAACT (357-386)  amplicon size: 0.42 kb | (94^o^C- 30 sec,  70^o^C- 45 sec,  72^o^C- 2 min)  37 cycles | (Chen et al., 2008) |
| NPT II F’  NPT II R’ | GAGGCTATTCGGCTATGACTG (54-74)  ATCGGGAGCGGCGATACCGTA (773-753)  amplicon size: 0.7 kb | (95^o^C- 45 sec,  60°C- 30 sec,  72°C- 1 min)  37 cycles | (Tian et al., 2009) |
| NPT II F’  NPT II R’ | CACGACGGGCGTTCCTTGC (216-234)  GGTGGTCGAATGGGCAGGTAGC  (388-409)  amplicon size: 0.2 kb | (95°C-45 sec,  65°C- 30 sec,  72°C- 1 min)  37 cycles | (Zhang et al., 2015) |

Chen QF, Xiao S, Chye ML. Overexpression of the Arabidopsis 10-kilodalton acyl-coenzyme A-binding protein ACBP6 enhances freezing tolerance. Plant Physiol. 2008;148(1):304-15.

Tian L, Canli FA, Wang X, Sibbald S. Genetic transformation of Prunus domestica L. using the hpt gene coding for hygromycin resistance as the selectable marker. Scientia Horticulturae. 2009;119(3):339-43.

Zhang M, Zhuo X, Wang J, Wu Y, Yao W, Chen R. Effective selection and regeneration of transgenic sugarcane plants using positive selection system. In Vitro Cellular & Developmental Biology-Plant. 2015;51(1):52-61.
